# Supplementary material for: Long time-scales in primate amygdala neurons support aversive learning
Source: Nat Commun. 2018 Oct 26;9:4460. doi: 10.1038/s41467-018-07020-4 (PMC6203797; doi:10.1038/s41467-018-07020-4)
Supplement: Supplementary file 1 — Supplementary Information [file 41467_2018_7020_MOESM1_ESM.pdf]

Long time-scales in primate amygdala neurons support aversive learning

Aryeh H. Taub et al.

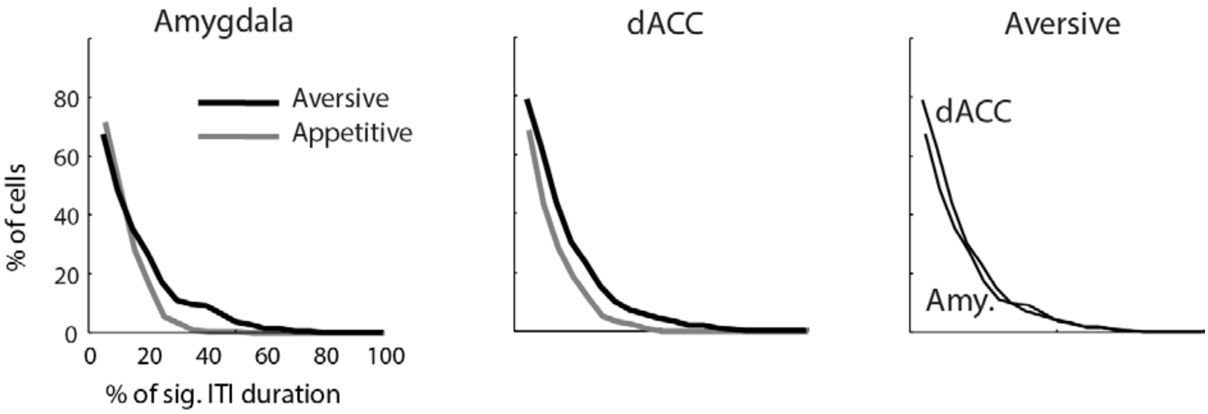

Supp.Fig.1. Proportions of cells with ITI-FR modulations.

Similar to Fig.3E. Histograms of the proportion of cells (y-axis) that showed significant ITI-FR modulation (ITI-FR > 2 s.t.d of the shuffled FR) in a proportion of the ITI duration (x-axis). All histograms are significantly different than a null distribution obtained from shuffling (Fig.3E).

Left: In the amygdala proportion of cells in aversive learning was slightly higher than in appetitive learning.

Middle: Similarly in the dACC.

Right: The proportion of cells in aversive learning was similar in the amygdala and the dACC.

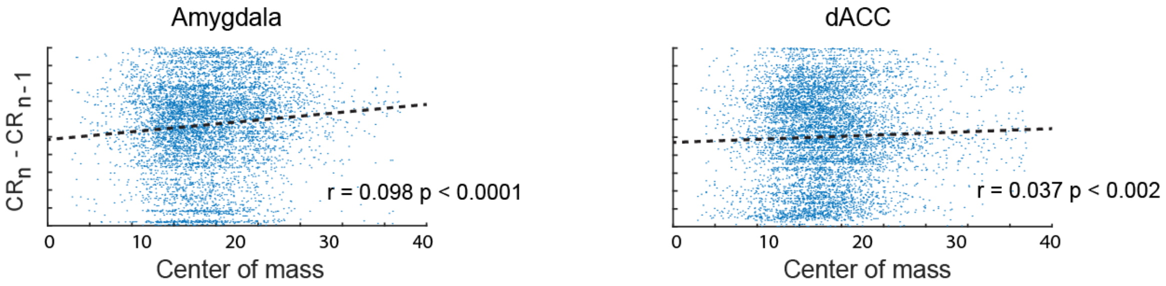

Supp.Fig.2. Temporal characteristics of ITI neural activity correlate with the behavioral gain (learning).

Similar to Fig.5C separately for each brain region: Change in CR amplitude between successive trials plotted against the center-of-mass of neural activity in the ITI between the trials. Left: Amygdala; Right: dACC. Shown are all single-units in all trials, and the corresponding linear regression. n_{days} of behavioral sessions = 82, Single units: amygdala: n = 291; dACC: n = 263.

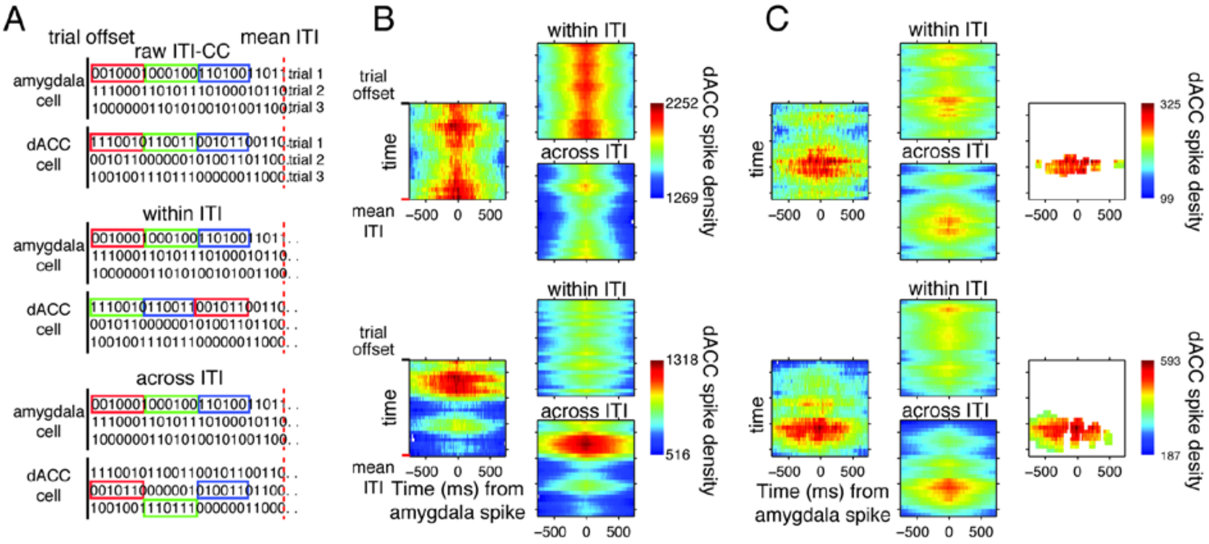

Supp.Fig.3. Quantifying amygdala-dACC pairwise cross-correlations (CC) along the ITI

Cross-correlations were computed between simultaneously recorded amygdala-dACC neurons in sliding 3-seconds segments of the ITI. All CCs are triggered on amygdala spike (at time zero). CCs were compared to shuffles to exclude changes resulting from covariations within and across ITIs. All maps show the time in the ITI on y-axis (top-to-bottom), and the CC itself as time from amygdala spike on the x-axis, colored by spike-density.

(A) To identify the significant correlation bins we compared the raw CC (left in B,C) against two types of shuffled cross-correlations (A, and right maps in B,C). (1) within-ITI shuffle (A, middle column) exchange regions within each ITI, and removes correlations that stem from covariance in the FR along the ITI. (2) across-ITI shuffle (A, lower column) exchange the same temporal regions in the ITI across different trials, and removes correlations that stem from covariance in the FR along the trials.

(B) Shown are two examples where only one shuffle, but not the other, account for the observed raw cross-correlations. Such CCs were not deemed significant.

(C) Shown are two examples where neither of the shuffled cross correlations account for the raw cross correlation. Maps on the right show the bins deemed significant after comparison to the two shuffles.

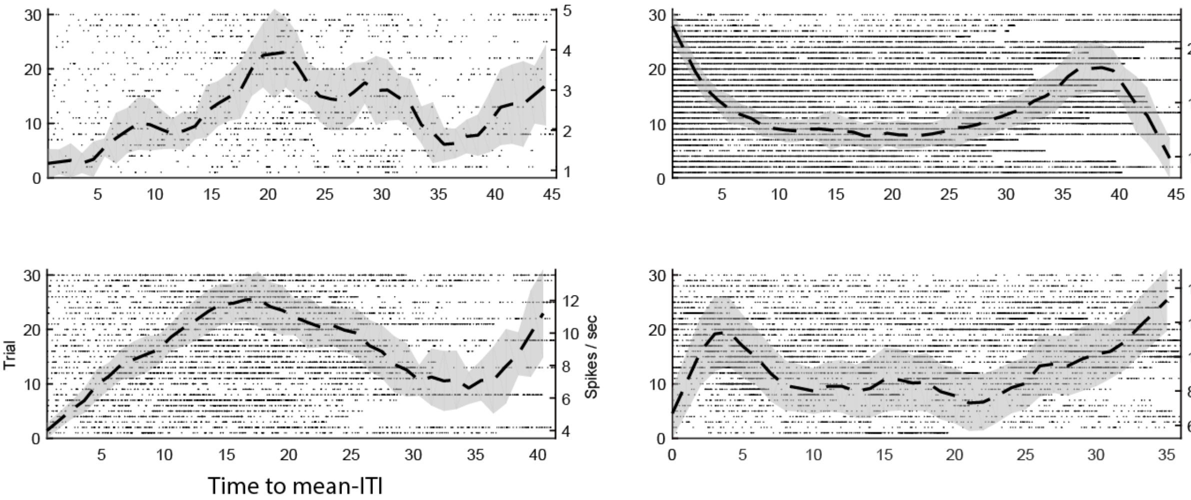

Supp.Fig.4. Single-cell examples during the ITI

Four examples of raster plots overlaid by PSTH (mean firing rate ± SEM).
Two amygdala cells (left column) and two dACC cells (right column). Activity is presented up to the mean ITI duration in the session.

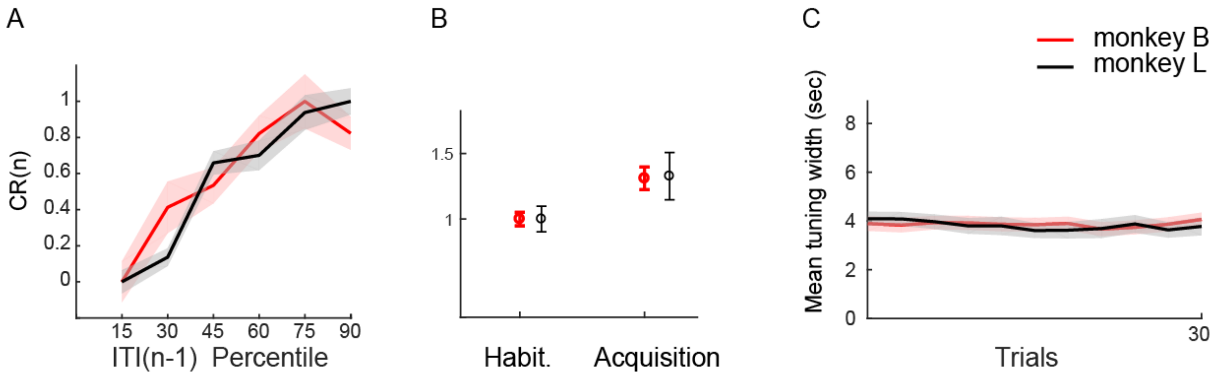

Supp.Fig.5. Learning, unit modulation, and tuning width for the two animals

(A) CR amplitude plotted against ITI duration in the previous trial (as in Fig.1A), separately for each monkey.

(B) Increase in mean RMS during conditioning occurs in the two monkeys, averaged over all neurons.

(C) Mean tuning-width over all single-units during the learning trials (as in Fig.6C-middle;); shown separately for the two animals.

Data presented as mean ± SEM.
